# Levofloxacin loaded poly (ethylene oxide)-chitosan/quercetin loaded poly (D,L-lactide-co-glycolide) core-shell electrospun nanofibers for burn wound healing

**DOI:** 10.3389/fbioe.2024.1352717

**Published:** 2024-03-28

**Authors:** Mahshid Monavari, Razieh Sohrabi, Hamidreza Motasadizadeh, Mehran Monavari, Yousef Fatahi, Negin Mousavi Ejarestaghi, Miguel Fuentes-Chandia, Aldo Leal-Egaña, Mohammad Akrami, Shahin Homaeigohar

**Affiliations:** ^1^ Department of Pharmaceutical Biomaterials and Medical Biomaterials Research Center, Faculty of Pharmacy, Tehran University of Medical Sciences, Tehran, Iran; ^2^ Dental Research Center, Dentistry Research Institute, Tehran University of Medical Sciences, Tehran, Iran; ^3^ Section eScience (S.3), Federal Institute for Materials Research and Testing, Berlin, Germany; ^4^ Nanotechnology Research Centre, Faculty of Pharmacy, Tehran University of Medical Sciences, Tehran, Iran; ^5^ Department of Pharmaceutical Nanotechnology, Faculty of Pharmacy, Tehran University of Medical Sciences, Tehran, Iran; ^6^ Department of Pharmaceutics, Faculty of Pharmacy, Tehran University of Medical Sciences, Tehran, Iran; ^7^ Department of Biology, Skeletal Research Center, Case Western Reserve University, Cleveland, OH, United States; ^8^ Institute for Molecular Systems Engineering and Advanced Materials, Heidelberg University, Heidelberg, Germany; ^9^ Institute of Biomaterials, University of Tehran & Tehran University of Medical Sciences (IBUTUMS), Tehran, Iran; ^10^ School of Science and Engineering, University of Dundee, Dundee, United Kingdom

**Keywords:** core-shell nanofiber, co-axial electrospinning, drug delivery, burn wound, wound healing

## Abstract

This study developed a new burn wound dressing based on core-shell nanofibers that co-deliver antibiotic and antioxidant drugs. For this purpose, poly(ethylene oxide) (PEO)-chitosan (CS)/poly(D,L-lactide-co-glycolide) (PLGA) core-shell nanofibers were fabricated through co-axial electrospinning technique. Antibiotic levofloxacin (LEV) and antioxidant quercetin (QS) were incorporated into the core and shell parts of PEO-CS/PLGA nanofibers, respectively. The drugs could bond to the polymer chains through hydrogen bonding, leading to their steady release for 168 h. An *in vitro* drug release study showed a burst effect followed by sustained release of LEV and QS from the nanofibers due to the Fickian diffusion. The NIH 3T3 fibroblast cell viability of the drug loaded core-shell nanofibers was comparable to that in the control (tissue culture polystyrene) implying biocompatibility of the nanofibers and their cell supportive role. However, there was no significant difference in cell viability between the drug loaded and drug free core-shell nanofibers. According to *in vivo* experiments, PEO-CS-LEV/PLGA-QS core-shell nanofibers could accelerate the healing process of a burn wound compared to a sterile gauze. Thanks to the synergistic therapeutic effect of LEV and QS, a significantly higher wound closure rate was recorded for the drug loaded core-shell nanofibrous dressing than the drug free nanofibers and control. Conclusively, PEO-CS-LEV/PLGA-QS core-shell nanofibers were shown to be a promising wound healing material that could drive the healing cascade through local co-delivery of LEV and QS to burn wounds.

## 1 Introduction

Wounds can be classified in different types depending on their cause or origin, e.g., incision, abrasion, puncture, burns, etc. (Abid et al., 2018). The burn wounds are a critical medical state and a global burden that needs to be treated and monitored on a regular basis (Singh et al., 2022; Sen et al., 2023). World Health Organization (WHO) has estimated the occurrence of 11 million burn injuries per year globally, 180,000 of which can lead to death (Jeschke et al., 2020). Burn injuries induce inflammatory responses and metabolic changes that can evoke the complications that are difficult to manage (Jeschke et al., 2020). Progressing to sepsis, infection is the main cause of death in the patients with burn injuries (Gomez et al., 2009; Nuutila et al., 2020). *Staphylococcus aureus* and *Pseudomonas aeruginosa* of gram-positive and gram-negative bacteria, respectively, are the most available pathogens in such wound beds that can cause infection (Norbury et al., 2016). Therefore, antimicrobial materials are essential in the treatment of infected burn wounds (Kumari et al., 2011). Levofloxacin (LEV) is a broad-spectrum antibiotic from the fluoroquinolone drug class that has been used in the treatment of burn wound infections. It offers a bactericidal effect via inhibition of bacterial DNA synthesis and further damage of DNA strands (Podder and Sadiq, 2022; Razdan et al., 2023). LEV has been shown to provide a high antibacterial activity against *S. aureus*, *P. aeruginosa*, *L. pneumophila*, and *salmonellae in vitro* (Smith et al., 2000). In addition to infection and inflammation, burn patients are vulnerable to ROS-mediated damages, thus, utilization of antioxidants can provoke the healing process of such wounds. Specifically, ROS plays a crucial role in the burn-induced suppression of immune system. In this regard, antioxidants can increase immune activity, thereby reducing the risk of burn wound infection (Al-Jawad et al., 2008; Sahib et al., 2010). Quercetin (QS) is a polyhydroxy flavonoid that is mainly found in flowers, leaves, and fruits of different plants (Fu et al., 2020). QS’ pharmacological activities include antioxidant, anti-inflammatory, and antimicrobial effects (Moskwik et al., 2023). QS’ antioxidant mechanism of action is based on its impact on Glutathione, enzymatic activity, signal transduction pathways, and the ROS generation driven by environmental and toxicological factors. As a result, QS can maintain the oxidative balance in the body (Xu et al., 2019). Although much research has been conducted on LEV and QS individually, their synergetic effect on chronic, infectious wounds has rarely been investigated. It has been shown that a more potent healing efficiency can be achieved by co-delivery of various therapeutic agents (Rezaei et al., 2020). In this regard, advanced wound dressings capable of reducing both bacterial infection and inflammation are appealing for burn wound treatment (Yin et al., 2022). For instance, Amani et al. fabricated a bilayer electrospun wound dressing containing gentamicin (antibiotic) and diclofenac (non-steroidal anti-inflammatory drug) for burn wound treatment. The nanofiber dressing with dual delivery of gentamicin and diclofenac was shown to offer an enhanced wound healing efficiency in an animal study (Amani et al., 2023a). Similarly, it is postulated that in the current study co-delivery of LEV and QS can lower the bacterial load of burn wounds and reduce inflammation, thereby cooperatively improving the wound healing conditions.

**FIGURE 1:**
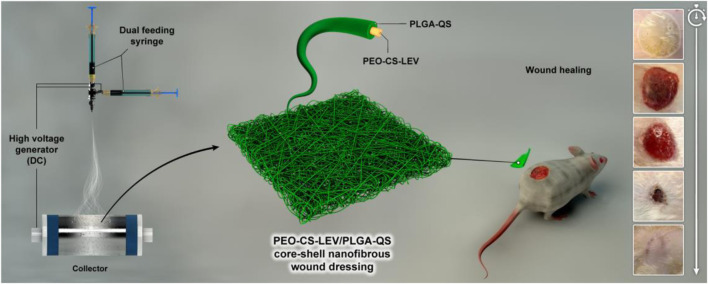
Schematic illustration of the preparation process and *in vivo* wound healing efficiency of PEO-CS-LEV/PLGA-QS core-shell nanofibrous wound dressing.

Among the various classes of drug delivery systems, nanofibers have attracted much attention due to their large available surface area, high porosity, and promising drug loading capacity (Cai et al., 2012; Homaeigohar and Boccaccini, 2020; Amani et al., 2023b). Electrospinning is a standard approach for fabrication of micro- and nanofibers (Reddy et al., 2016; Darbasizadeh et al., 2018; Homaeigohar et al., 2021; Selim et al., 2023). The nanofibrous materials show crucial advantages for drug delivery such as controlled, localized release of drugs and promising physicochemical properties, e.g., a high aspect ratio, a small diameter, and an extensive surface area that could be chemically engineered depending on the application (Luraghi et al., 2021). There is a large number of electrospun nanofibrous systems for drug delivery into wound beds. For instance, Ren et al. (Ren et al., 2018) devised an aligned porous fibrous membrane made of poly (l-lactic acid) (PLLA) reinforced with dimethyloxalylglycine (DMOG) loaded mesoporous silica nanoparticles. The co-delivery of DMOG and silicon ions by the PLLA fibers led to improved vascularization in a diabetic wound bed. As an advanced derivative of electrospinning, co-axial electrospinning enables the development of core-shell nanofibers with controlled drug release (Kaviannasab et al., 2019; Darbasizadeh et al., 2021). In the current study, we aim to fabricate a core-shell nanofibrous wound dressing made of chitosan (CS)-polyethylene oxide (PEO) blend as core and poly (lactic-co-glycolic acid) (PLGA) shell that co-delivers LEV and QS to burn wounds. Chitosan (CS) is a biodegradable natural polymer which offers an anti-inflammatory and antimicrobial activity (Vega-Cázarez et al., 2018; Sapkota and Chou, 2020). This biopolymer is largely used in drug delivery, tissue engineering, and wound healing (Yadollahi et al., 2016; Darbasizadeh et al., 2018; Salazar-Brann et al., 2021; Farhadnejad et al., 2022). The CS wound dressings accelerate wound healing and reduce pain and infection in burn wounds (Hu et al., 2023). Nevertheless, due to CS’ polycationic nature and inter/intra-molecular interactions, electrospinning of CS is challenging. To address this shortcoming, CS is blended with PEO to synthesize nanofibrous scaffolds (Yuan et al., 2016; Varnaitė-Žuravliova et al., 2020). PLGA is a biodegradable synthetic polymer widely used as a drug carrier with sustained drug release, optimum mechanical strength, and an appropriate degradation rate (Chereddy et al., 2016). Lactate, as a byproduct of PLGA degradation, can promote wound healing via enhanced angiogenesis, collagen synthesis, and endothelial progenitor cells recruitment (Chereddy et al., 2013; Chereddy et al., 2015). Cooperatively, PEO-CS/PLGA core-shell nanofibers can not only provide a biomimetic nanofibrous structure as seen in native skin tissue with collagen nanofibers, but also release therapeutic compounds such as LEV and QS in a sustained manner into a burn wound bed. As a result, an improved wound healing behavior is assumed to be achieved with such a sophisticated nanobioformulation and nanostructured wound dressing. Figure 1 schematically depicts the concept of our research based on co-electrospinning of PEO-CS-LEV/PLGA-QS core-shell nanofibers that could promote wound healing *in vivo*.

## 2 Materials and methods

### 2.1 Materials

Chitosan (76% deacetylated, and viscosity 122 cps, 1 wt% in 1% acetic acid), poly (ethylene oxide) (PEO, average Mv∼900,000), poly (D,L-lactide-co-glycolide) (PLGA, lactide: glycolide 50:50, Mw∼ 45 kg/mol), QS, and LEV were purchased from Sigma-Aldrich (Germany). N-methyl-2-pyrrolidone (NMP), dimethylformamide (DMF), and tetrahydrofuran (THF) were obtained from Merck (Germany). Bi-distilled water was used to prepare aqueous solutions. All reagents and chemicals were mainly of analytical grade and used as received without any further purification.

### 2.2 Preparation of core-shell nanofibers

The core PEO-CS-LEV solution was prepared by dissolving 150 mg CS (3% w/v) and 100 mg PEO (2% w/v) in 5 mL acetic acid aqueous solution (90% w/v). Subsequently, a given amount of LEV (10% w/w relative to the PEO-CS mass) was added to the above solution. The as-prepared solution was thoroughly stirred to get homogenized. To prepare the shell PLGA-QS solution, 1 g PLGA (20% w/v) and 50 mg QS (5% w/w relative to PLGA mass) were dissolved in 5 mL DMF/THF (2/1) and vigorously stirred.

To fabricate the PEO-CS-LEV/PLGA-QS core-shell nanofibers, the PEO-CS-LEV and PLGA-QS solutions were poured separately into two 5 mL plastic syringes connected to the coaxial spinneret of a co-axial electrospinning set-up (Fanavaran Nano-meghyas, Iran) and were electrospun under optimized electrospinning conditions including the collecting distance of 15 cm, applied voltage of 15 kV, and feed rate of 0.6 mL/h (core solution) and 1 mL/h (shell solution).

### 2.3 Physicochemical characterization

The morphology of PEO-CS-LEV/PLGA-QS core-shell nanofibers was imaged by a KYKY-EM3200 digital scanning electron microscope (SEM) after coating them with a thin Au layer under high vacuum at the acceleration voltage of 26 kV. The ImageJ software (version 1.52) was employed to quantify the diameter and diameter distribution of the core-shell nanofibers. Transmission electron microscope (TEM, Zeiss -EM10C) was used to visualize the core-shell structure of the nanofibers. For this purpose, the core-shell nanofibers were collected on a carbon-coated copper grid and TEM images were captured under the acceleration voltage of 80 kV. The physicochemical interactions of the various components of the PEO-CS-LEV/PLGA-QS core-shell nanofibers were investigated by using a FTIR spectrophotometer (BRUKER TENSOR 27) in the spectral range of 500–4,000 cm^−1^ at the resolution of 4.0 cm^−1^. Thermal gravimetric analysis (TGA) of the nanofibers was carried out by using a TGA-50H thermogravimetric device. To do this, 10 mg of the nanofibers was placed within the sealed aluminum pans that were heated up to 600°C at the heating rate of 10°C min^−1^ under a 20 mL min^−1^ nitrogen gas flow. STOE-STADI powder X-ray diffractometer was used to analyze the crystallinity of the nanofibers in the 2θ range of 5°–50°, under 40 kV and 40 mA, with Cu-Kα (λ = 1.54060 °A) radiation.

The mechanical properties of the core-shell nanofibers were determined through a uniaxial tensile test by using an Instron 5,566 tensile machine at ambient temperature. To do this, the nanofibrous mats were cut into rectangular specimens (0.5 cm × 3 cm) and stretched. From each class of the core-shell nanofibrous mats, 3 samples were tested.

### 2.4 *In vitro* drug release analysis

*In vitro* drug (LEV and QS) release rate of the PEO-CS-LEV/PLGA-QS core-shell nanofibrous mats was quantified at pH 7.4 and 37°C. Due to the overlap of LEV’s and QS’ UV-Vis spectra, simultaneous analysis of their release rate is impractical. Therefore, PEO-CS-LEV/PLGA and PEO-CS/PLGA-QS core-shell nanofibers’ drug release rates were separately characterized. To do this, three sections (3 × 3 cm^2^) of the core-shell nanofibers containing LEV and QS were precisely weighed and immersed in 10 mL PBS (pH 7.4) at 37°C under shaking (100 rpm) for 168 h. At regular time intervals, 2 mL of the supernatant was removed for UV-vis spectrophotometry at λ = 292 nm and 380 nm corresponding to LEV and QS, respectively, and the solution was replenished.

### 2.5 Drug release kinetics measurement

To assess the LEV and QS release kinetics, the *in vitro* release profile of the PEO-CS-LEV/PLGA-QS core-shell nanofibrous mats in PBS (pH 7.4) was fitted into different kinetic models (Fatahi et al., 2021):

$$f_{t}=k_{0}t$$ (1)

(Zero order kinetics model)

$$\ln\left(1-\;f_{t}\right)=k_{1}t$$ (2)

(First order kinetics model)

ft=kHt12 (3)

(Higuchi kinetics model)

$$f_{t}=k_{p}t^{n}\left(\ln f_{t}=\ln k_{p}+nlnt\right)$$ (4)

(Korsmeyer–Peppas kinetics model)

In Eqs 1–4, f_t_, t, n and k_P_ represent the fraction of drug released at time t, release time, release exponent, and rate constant, respectively. k_0_, k_1_ and k_H_ are the rate constants of the zero order, first order, and Higuchi models, respectively. n identifies the drug release mechanism. For cylindrical compounds, n ≤ 0.45, 0.45 < n < 1, n = 1, and n > 1 are indicative of the Fickian diffusion release, non-Fickian diffusion release, Case-II transport or zero-order kinetics, and supper case-II transport, respectively (Fatahi et al., 2021).

### 2.6 Cell viability assay

NIH 3T3 fibroblast cell viability of the PEO-CS-LEV/PLGA-QS core-shell nanofibers was investigated through the 3-(4,5-dimethylthiazol-2-yl)-2,5-diphenyltetrazolium bromide (MTT) colorimetric assay. This cell line is of importance due to its decisive role in the regeneration of connective tissues and in the reconstruction of ECM. The Dulbecco’s Modified Eagle Medium (DMEM) was used as the culture medium which was supplemented with penicillin and streptomycin (1%) as antimicrobial agents and FBS (10%) as nutrient. The cell containing medium was incubated for 72 h under 5% CO_2_/95% air at 37°C. Afterwards, 100 μL NIH 3T3 fibroblast cells with the density of 1×10^4^ cells/well was incubated in a 96-well plate for 24 h under the same atmospheric condition. The extract of the core-shell nanofibers was UV irradiated for 1 h and then incubated in the culture medium for 24 h at 37°C. Thereafter, NIH 3T3 fibroblast cells were subjected to the extracts for 24, 48, and 72 h. At each time point, 10 μL MTT reagent was incubated with the cell-extract assemblies for 3 h. Subsequently, 100 µL DMSO was added to the medium and the assembly was shaken for 10 min to dissolve the purple-colored formazan crystals. Eventually, the medium was optically analyzed at λ = 570 nm using an Epoch microplate reader (Bio-Rad, model 550).

### 2.7 *In vivo* wound healing efficiency measurement

All animal experiments were carried out in full compliance with the guidelines approved by the ethics committee of Tehran University of Medical Sciences (approval No. IR.TUMS.PSRC.REC.1396.4146). Wistar albino rats (male, weight = 200–250 g) were divided in 5 groups of: 1) Control, 2) PEO-CS/PLGA, 3) PEO-CS/PLGA-QS, 4) PEO-CS-LEV/PLGA, and 5) PEO-CS-LEV/PLGA-QS. Each group consisted of 7 rats with access to standard food and water. First, the rats were anesthetized in a ratio of 80 to 20 by intraperitoneal injection of ketamine hydrochloride (50 mg/kg) and xylazine (5 mg/kg). After dorsal hair removal, 20 mm thermal burn wounds were made by direct contact of skin with a hot aluminum rod (110°C) for 5 s. The as-formed wounds were deep enough to resemble the second degree burn wounds. The burn wounds were immediately treated with the nanofibrous wound dressings and a sterile gauze in the control group.

The wound closure rate was determined based on digital images of the wounds captured on days 3, 7, 14, and 21 post-treatment. To measure the wound size (area), the images were analyzed using the ImageJ software and wound closure rates were quantified via equation 5:

$$Wound\;closure\;rate\;\left(\%\right)=\frac{\left(Wound\;area\;on\;day\;0\right)-\left(Wound\;area\;on\;days\;3,7,14\;and\;21\right)}{\left(Wound\;area\;on\;day\;0\right)}\times 100$$ (5)

### 2.8 Histological analysis

To carry out histological analysis, the rats underwent euthanasia through intraperitoneal injection of ketamine (300 mg/kg) and xylazine (20 mg/kg) and wound tissues were completely excised on days 7, 14 and 21 post-treatment. The collected wound tissues were fixed by immersion in a 10% formalin buffer solution for 48 h and further embedded within paraffin wax. Thereafter, the samples were sectioned into 5 µm thick slices and stained through Haematoxylin and eosin (H&E) and Masson’s trichrome (MT) staining assays. Ultimately, an independent pathologist assessed the histological slides using a light microscope (Olympus, Japan) under 40x and 100x magnifications.

### 2.9 Statistical analysis

The cell test and *in vivo* (wound closure percentage) data were analyzed statistically through the one-way analysis of variance (ANOVA) technique. It is worth mentioning that all measurements were repeated thrice, and the obtained values were reported as mean ± standard deviation.

## 3 Results and discussion

### 3.1 Physicochemical characteristics of the PEO-CS-LEV/PLGA-QS core-shell nanofibers

**FIGURE 2:**
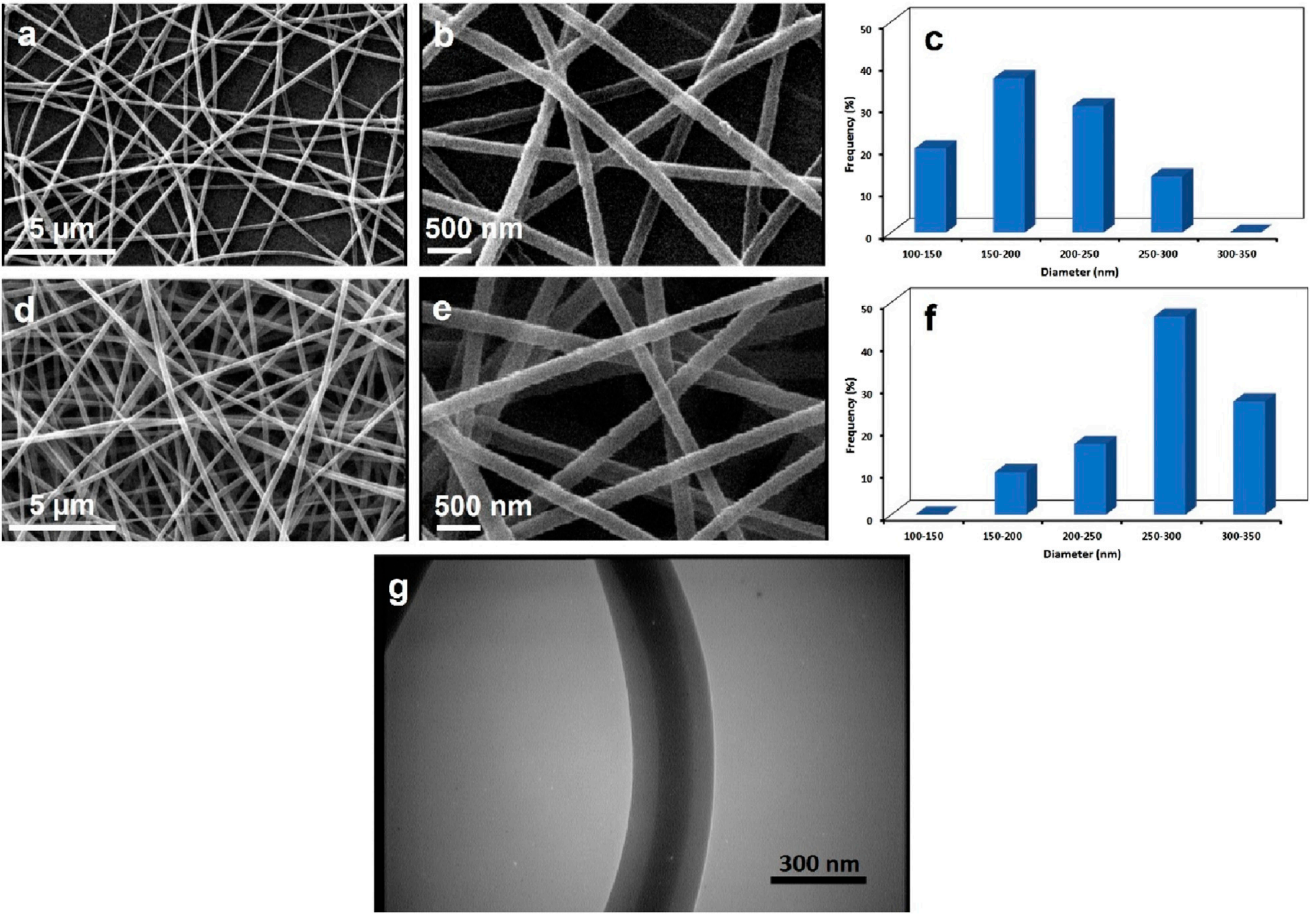
SEM images and diameter histograms of PEO-CS/PLGA **(A–C)** and PEO-CS-LEV/PLGA-QS **(D–F)** core/shell nanofibers. The images indicate a uniform nanofiber diameter distribution with no beads for the core-shell nanofibers w and w/o drugs. **(G)** TEM image of a PEO-CS-LEV/PLGA-QS core/shell nanofiber which obviously visualizes two distinct phases in the core and shell parts, implying the immiscibility of the PLGA-QS and CS-PEO-LEV solutions during electrospinning.

The SEM images of PEO-CS/PLGA and PEO-CS-LEV/PLGA-QS core-shell nanofibrous mats (Figure 2A,B,D,E) show that the nanofibers w and w/o the drugs feature a uniform diameter distribution and are bead-less. The average diameter of PEO-CS/PLGA and PEO-CS-LEV/PLGA-QS core/shell nanofibers (quantified using the ImageJ software) was 190 ± 50 nm and 269 ± 50 nm, respectively. There is a statistically significant difference in the average fiber diameter of these two classes of core/shell nanofibers (*p* < 0.05). The incorporation of LEV and QS in the core and shell sections of the nanofibers significantly increases their respective diameter (Figure 2C,F), as a result of enhanced polymer solutions’ viscosity. The possible physicochemical bonding between ether and amine groups of PEO and CS, respectively, with LEV’s C-N, C-F, C=O, and OH groups (as will be discussed later) could raise the viscosity of the PEO-CS-LEV solution. On the other hand, ether and carbonyl groups of PLGA could form a hydrogen bond with the hydroxyl groups of QS and similarly increase the viscosity of the PLGA-QS solution.

Figure 2G shows a TEM image the PEO-CS-LEV/PLGA-QS core-shell nanofibers wherein core and shell regions are visually distinct. The light and dark regions represent the PEO-CS-LEV and PLGA-QS phase, respectively, indicating the successful formation of a core/shell structure in the nanofibers derived from immiscibility of the polymer solutions during electrospinning. The presence of CH_3_ side groups in poly(lactide acid) (PLA) chains endows a superior hydrophobicity to this polymer relative to poly(glycolic acid) (PGA) (Makadia and Siegel, 2011). Therefore, the lactide rich PLGA (i.e., copolymer of PLA and PGA) is almost hydrophobic (Makadia and Siegel, 2011) and poorly soluble in polar solvents such as acetic acid (i.e., the solvent of PEO-CS solution). On the other hand, PEO and CS are inadequately soluble in DMF/THF, thus PEO-CS/acetic acid solution remains immiscible with PLGA solution at the onset of co-axial electrospinning in a very short time frame, particularly at room temperature.

With respect to the formation mechanism of the core-shell nanofibers, it is assumed that the PLGA shell solution assists to the electrospinning of less-electrospinnable CS-PEO core solution. Over the course of the coaxial electrospinning process, the PLGA shell solution drags the CS-PEO core solution to form a stable compound Taylor cone and later a continuous jet. This behavior might be ascribed to the higher conductivity of the shell solution (Pakravan et al., 2012). While CS solution is a polyelectrolyte with optimum electrical conductivity and PEO solution is neutral (Pakravan et al., 2012), blending of CS and PEO solutions leads to loss of the CS solution conductivity. On the other hand, PLGA’s functional group can be ionized in the shell solution during electrospinning and improve the conductivity of the solution. According to Yu et al. (Yu et al., 2004), the higher shell solution conductivity compared to the core solution’s can potentially stabilize the coaxial electrospinning process, most likely due to a higher extent of shear stress that is applied on the core solution and a larger resulting stretching force, leading to formation of a thinner core.

**FIGURE 3:**
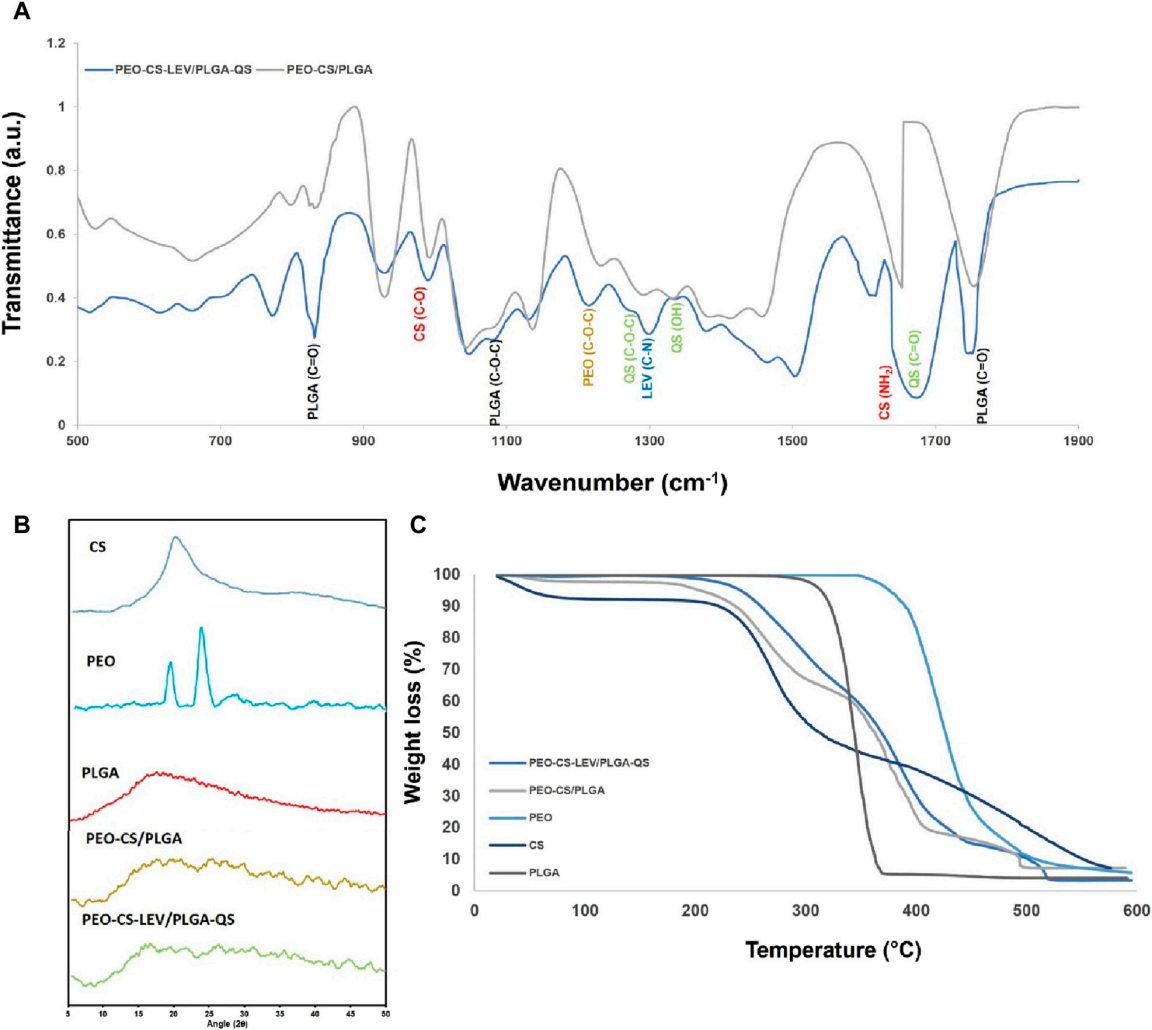
**(A)** FTIR spectra, **(B)** XRD spectra, and **(C)** TGA profile of PEO-CS-LEV/PLGA-QS nanofibers and their core and shell phases. The FTIR spectra clearly imply the physicochemical interaction of PEO and CS with LEV and PLGA with QS through hydrogen bonding, reflected in the band shifts and band intensity loss. The core-shell nanofibers are mainly amorphous with no distinct crystalline peaks. Semi-crystalline PEO is amorphized when blended with CS and LEV. Compared to the core-shell nanofibers w/o drugs, improved thermal stability is seen for the core-shell nanofibers containing the drugs, indicating intermolecular bonding of the polymer chains mediated by the drug molecules.

The physicochemical interactions of the components of the core-shell nanofibers were tracked through FTIR spectroscopy. As seen in Figure 3A, FTIR spectra of the core-shell nanofibers w and w/o the drugs feature several characteristic bands of PLGA at 1750 cm^−1^ (C=O), 1,088 cm^−1^ (C–O–C), and 827 cm^−1^ (C=O) (Haider et al., 2016; Tohidi et al., 2016). Upon addition of QS to the core-shell nanofibers, new bands corresponding to QS’ oxygen bearing groups appear at 1,676 cm^−1^, 1,337 cm^−1^, and 1,283 cm^−1^ that represent C=O, C-OH, and ether stretching vibration, respectively. Compared to pristine QS, such bands have shifted from 1,665 cm^−1^ (C=O), 1,317 cm^−1^ (C–OH), and 1,261 cm^−1^ (ether) (Bukhari et al., 2008), most likely due to hydrogen bonding between these functional groups and those of PLGA (e.g., between OH and C=O). Similarly, Anwer et al.(Anwer et al., 2016) have reported a band shift of QS entrapped within PLGA nanoparticles. Supplementary Figure S1A shows the FTIR spectrum of pristine PLGA nanofibers wherein the characteristic bands of PLGA are located at 870 cm^−1^(C=O), 1,094 cm^−1^ (ether), and 1778 cm^−1^ (C=O). The band shift for the nanofibers containing QS compared to the pristine PLGA nanofibers clearly indicates hydrogen bonding between QS and PLGA. On the other hand, compared to the core-shell nanofibers w/o QS (and pristine PLGA nanofibers shown in Supplementary Figure S1A), intensity of the PLGA bands notably declines, implying a significant interaction between PLGA and QS.

The FTIR spectrum of CS nanofibers (Supplementary Figure S1B) shows several transmittance bands representing electron donating groups at 3,200–3,450 cm^−1^ (OH/NH), 1,633 cm^−1^ (C-O stretching of the acetyl group (amide I)), 1,255 cm^−1^ (OH), and 1,066 cm^−1^ (C-O) (Choo et al., 2016). PEO’s FTIR spectrum also features two main transmittance bands of ether groups at 1,151 cm^−1^ and 1,032 cm^−1^(Lin et al., 2018). The FTIR spectrum of CS-PEO blend as reflected in that of the core-shell nanofibers w/o drugs (Figure 3A) shows one transmission band at 1,653 cm^−1^ corresponding to the amine group of CS (Hussein-Al-Ali et al., 2018) and PEO’s C-O band at 1,138 cm^−1^(Ibrahim et al., 2023). Compared to pristine CS and PEO nanofibers, amine and ether bands have shifted most likely due to hydrogen bonding between the mentioned groups (Hussein-Al-Ali et al., 2018; Ibrahim et al., 2023). With incorporation of LEV, as seen in the FTIR spectrum of the core-shell nanofibers with the drugs (Figure 3A), a new band appears at 1,300 cm^−1^ that represents C-N group of LEV. The other characteristic bands of LEV such as those at 3,433 cm^−1^, 1730 cm^−1^, and 1,080 cm^−1^ that could represent O–H, C=O, and C-F stretching vibrations of LEV, respectively, are hidden under the transmittance bands of PLGA, CS, and PEO (Bandari et al., 2017; Islan et al., 2017). It turns out that the original transmittance bands of CS and PEO further shift after addition of LEV. For instance, PEO’s ether band at 1,138 cm^−1^ shifts to 1,130 cm^−1^ and CS’ amide band at 1,653 cm^−1^ shifts to 1,639 cm^−1^. Additionally, the intensity of such bands drastically declines.

Figure 3B shows the XRD spectra for the core-shell nanofibers w and w/o the drugs. While PLGA and CS feature a broad XRD band at the 2θ range of 10°–20°, indicating their amorphous nature (Hashemikia et al., 2021; Huang et al., 2021), the XRD spectrum of PEO possesses two sharp peaks at 2θ of 19.2° and 23.4°, representing the (120) and (112) crystallographic planes, respectively (Malwal and Gopinath, 2015). Regarding the incorporated drugs in pristine form, XRD spectra for QS and LEV (Supplementary Figure S2) show several characteristic peaks at 2θ_LEV_ = 6.6°, 9.7°, 13°, 15.7°, 19.4°, 26.3°, 31.5°, and 45.4° (Islan et al., 2017), and 2θ_QS_ = 10.7°, 12.3°, 16.0°, 23.6° and 27.1°(Patel et al., 2012). Despite the strong crystallinity of both QS and LEV in pristine form, they are amorphized upon combination with the polymers, most likely due to bonding with polymeric chains which inhibits their crystallization during the electrospinning process. Similarly, Patel et al (Patel et al., 2012) have reported that QS loaded on Zein colloidal particles turns amorphous due to its nanoscale confinement which challenges the crystallization process. Additionally, the formation of an amorphous assembly with proteins within the particle matrix (polymer chains in our study) can play a significant role in amorphization of the incorporated drugs. On the other hand, extensive intermolecular bonding of PEO with LEV and CS could be responsible of amorphization of PEO in the core-shell nanofibers w or w/o drugs. This behavior has been reported for the polysaccharide (dandelions) incorporated PEO nanofibers as well (Lin et al., 2018).

Figure 3C shows the TGA profile of PEO-CS/PLGA core-shell nanofibers w and w/o LEV and QS. Evidently, the core-shell nanofibers with drugs are more thermal resistant, thanks to the intermolecular bonding of the polymers with the incorporated drugs, that might even act as cross-linkers between the polymer chains. The onset of weight loss for the core-shell nanofibers with drugs takes place at 200°C, while this occurs sooner for the core-shell nanofibers w/o drugs at 55°C. The thermal decomposition temperature, i.e., the temperature at which 5% weight loss happens (S.Sh. Homaeigohar et al., 2012), was largely higher for the core-shell nanofibers with drugs (240°C) than those without drugs (203°C). Compared to the pristine polymers, i.e., CS, PLGA, and PEO, the core-shell nanofibers are degraded at lower temperatures. PEO as the most thermally stable polymer among the applied polymers is a semi-crystalline polymer. However, as discussed earlier when blended with CS, it turns amorphous, thus loses its high thermal stability. In the TGA profile of the core-shell nanofibers w and w/o drugs, the onset of PEO degradation is at 340°C, while in the pristine form, it degrades at 350°C. CS shows the lowest thermal stability and as a component of the core-shell nanofibers degrades at 190°C–328°C. As reported by Nam et al. (Nam et al., 2010), crystallinity and deacetylation degree of CS largely affects its thermal degradation temperature. The CS’ thermal degradation as blended with PEO in the core-shell nanofibers takes place at 190°C which is much lower than that reported by Nam et al. (272.8°C) (Nam et al., 2010) and Nista et al. (257°C) (Nista et al., 2015). While the CS’ deacetylation degree in our study is lower than theirs (76% vs. 85% for Nam et al.), a controversially lower thermal degradation temperature is recorded, likely due to the decreased crystallinity of CS after blending with PEO. Pristine PLGA nanofibers undergo a drastic weight loss after 335°C and the highest weight loss is seen at 367°C (Huang et al., 2021). As the shell part of the core-shell nanofibers, PLGA degrades at the temperature range of 339°C–409°C which overlaps with that of PEO. The relatively higher thermal stability of PLGA in the core-shell nanofibers with drugs compared to the pristine PLGA nanofibers could be attributed to the presence of QS and intermolecular bonding of QS and PLGA. Similarly, Guimaraes et al.(Guimarães et al., 2015) have reported a higher thermal stability for the PLGA nanofibers containing daunorubicin. The thermal behavior of LEV and QS are illustrated in Supplementary Figure S3.

**FIGURE 4:**
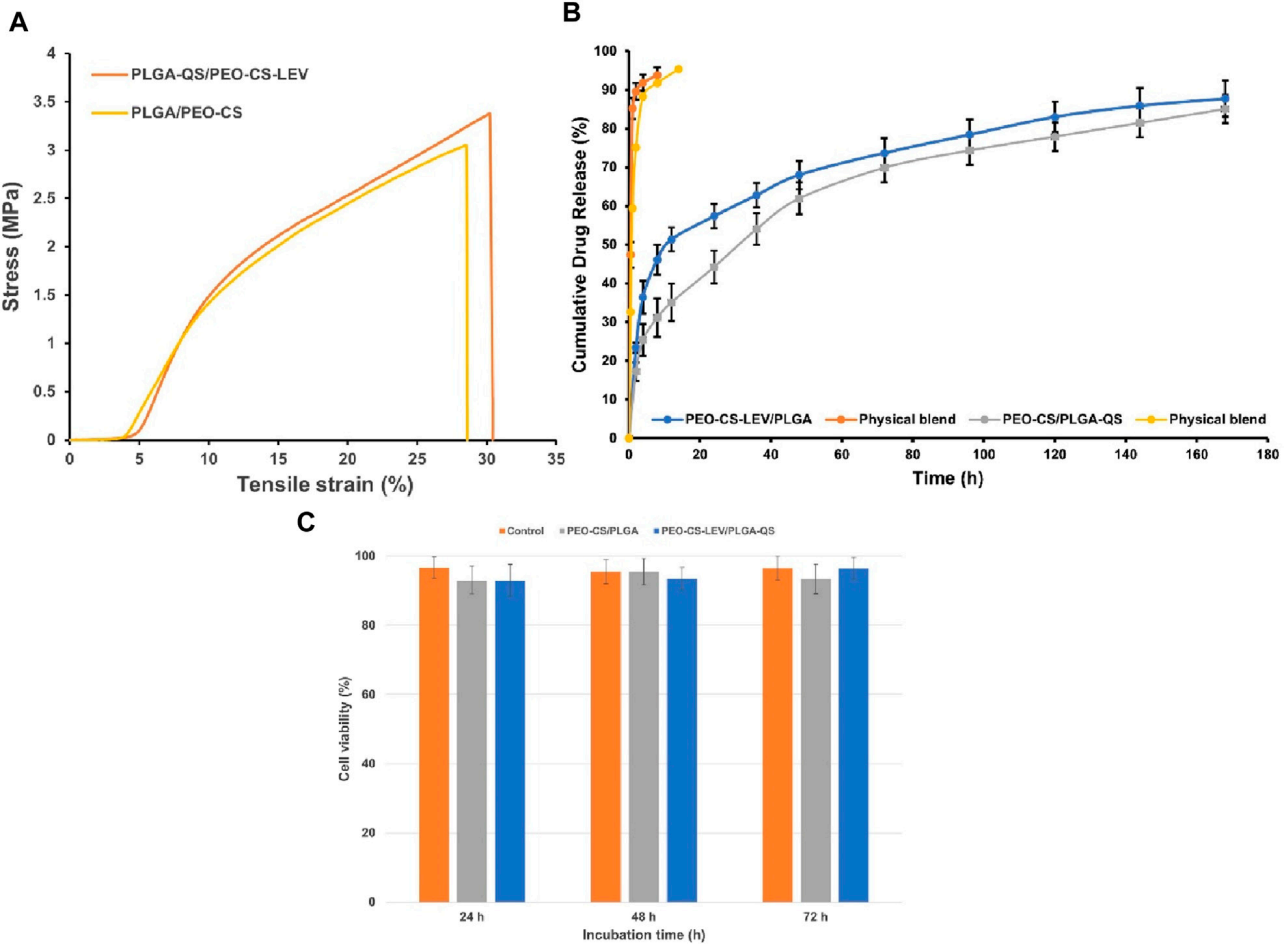
**(A)** Stress-strain graphs of the core-shell nanofibers w and w/o LEV and QS. A superior tensile strength, elongation, and toughness are observed for the drug loaded core-shell nanofibers that originates from intermolecular bonding of the drugs and polymers. It is worthy to note that elastic modulus of the core-shell nanofibers matches that of natural skin, thus providing comparable biomechanical signaling pathways. **(B)** *In vitro* drug release profile of PEO-CS-LEV/PLGA (d) and PEO-CS/PLGA-QS (e) core-shell nanofibers. The physical blends include PEO-CS-LEV and PLGA-QS, respectively. The core-shell nanofibers undergo a burst effect within the first 2 h immersion in PBS (likely due to the Coffee ring effect induced accumulation of LEV and QS on the surface), followed by a steady release until the end of the experiment (thanks to intermolecular bonding of the drug molecules and polymers). **(C)** NIH 3T3 fibroblast cell viability in the proximity of PEO-CS/PLGA and PEO-CS-LEV/PLGA-QS core-shell nanofibers. Both classes of the core-shell nanofibers (w and w/o drugs) support cell viability at a comparable level with the control. Noteworthy, there is no significant impact of the incorporated drugs on the cell viability.

The physicochemical interaction of the drugs and polymers was assumed to raise the resilience and mechanical strength of the core-shell nanofibrous mats. Figure 4A shows the stress-strain curves of the PEO-CS/PLGA core-shell nanofibers w and w/o LEV and QS. As clearly seen in this figure, the core-shell nanofibers with drugs are superior to their drug free counterparts in terms of tensile strength (3.4 MPa vs 3 MPa, i.e., 13% increment), elongation (30.4% vs 28.6%, i.e., 6.3% increment), and elastic modulus (0.33 MPa vs 0.25 MPa, i.e., 32% increment). As discussed earlier, such improved mechanical performance originates from intermolecular bonding of the drugs and polymers in the core and shell phases of the nanofibers. A high quality wound dressing is elastic and pliable, yet mechanically robust to protect the wounded tissue against further damage (Homaeigohar et al., 2023). The elastic modulus (mechanical stiffness) of a wound healing material largely affects the cellular activities, because cell-material interplay depends on the shear stresses imposed on the cells and on the mechanical signaling pathways that control the cell migration, proliferation, and differentiation (Stevens and George, 2005; Homaeigohar et al., 2020). Ideally, there should be a mechanical match between a wound dressing material and the skin tissue under treatment to provide comparable biomechanical signals (Homaeigohar et al., 2022). According to the literature (Lanno et al., 2020), elastic modulus of different classes of human skin (different origins) ranges from 8 kPa to 70 MPa. The elastic modulus of PEO-CS-LEV/PLGA-QS core-shell nanofibrous dressing is 0.33 MPa, that properly lies in this range. The mechanical properties of the different classes of the core-shell nanofibers are tabulated in Supplementary Table S1.

### 3.2 *In vitro* drug release behavior of the core-shell nanofibers

The nanofibrous meshes have been shown to perform as delivery carriers of bioactive materials (Mickova et al., 2012). Such a potential is justified by preservation of the bioactivity of the incorporated drugs and biomolecules and their sustained release in accordance with the tissue regeneration time frame (Ji et al., 2011). As seen in Figure 4B, the PEO-CS-LEV/PLGA and PEO-CS/PLGA-QS core-shell nanofibers demonstrate a burst release of ∼25% LEV and 16% QS in PBS within 2 h, followed by a sustained drug release over the next hours (up to 168th h). The burst release might be due to the initial swelling of the nanofibers and/or accumulation of the drug molecules on the nanofiber surface (QS) or at the interface of the core and shell phases (LEV) during electrospinning. Having a higher solubility in the solvents compared to the polymers, the coffee ring effect, which is a spontaneous hydrodynamic process (Chen et al., 2017), drives the drug molecules towards the surface during drying. After the initial burst release, the drugs are released steadily until an almost plateau (70%–80% release) is achieved at 80th h. Such a behavior is mainly attributed to the intermolecular bonding between the drug molecules and polymers, as explained earlier. At this stage, the drugs entrapped inside the core-shell nanofibers are released via a diffusional mechanism plus nanofibers degradation. Islan et al.(Islan et al., 2017) have reported a similar behavior for the LEV and DNase loaded CaCO_3_/alginate hybrid microparticles where the cargos are released in up to 6 h and then steadily for the rest of measurement (72 h).

To investigate the drug release kinetics, the release rate of LEV and QS from the PEO-CS-LEV/PLGA-QS core-shell nanofibrous mats was investigated according to the Korsmeyer-Peppas kinetic model. As tabulated in Table 1, for LEV and QS delivery, the n values are 0.32 and 0.38, respectively. Therefore, LEV and QS are released from the core/shell nanofibrous mats through the Fickian diffusion mechanism and under a concentration gradient between the nanofibers and external medium (PBS). As a matter of fact, swelling of glassy (amorphous) polymers, e.g., PLGA at the first hours of PBS immersion, involves the polymer chain relaxation at the swelling interface, thereby slowing the drug diffusion rate through the polymer. Such a situation that could lead to a steadier release is known as Stefan or Stefan-Neumann problem (Peppas and Narasimhan, 2014). The drug release data were fitted to the zero-order, first-order, and Higuchi kinetic models (Table 1). According to Table 1, the correlation coefficient ( $R_{h}^{2}$ ) of the Higuchi model is higher than that of other kinetic models ( $R_{0}^{2}$ and $R_{1}^{2}$ ) for both LEV and QS. This indicates that the kinetics data for the release of LEV and QS from the core-shell nanofibrous mats are in good agreement with the Higuchi kinetic model. According to this model, solvent gradually swells the matrix (PLGA and PEO-CS in our study), and a linear concentration gradient decreases from the saturation concentration at the interface with the core untouched by solvent, to concentration zero at the interface of matrix–dissolution medium (Mircioiu et al., 2019). The release constant of the Higuchi model (k_h_) for LEV and QS released from the core-shell nanofibrous mats are 8.14 and 7.80, respectively.

**TABLE 1:** Kinetic model parameters for LEV and QS released from the PEO-CS-LEV/PLGA-QS core-shell nanofibrous mats; n: kinetic exponent, *R* ^2^: regression coefficient.

	LEV	QS
N	0.322	0.379
$R_{0}^{2}$	0.777	0.961
$R_{1}^{2}$	0.866	0.988
$R_{h}^{2}$	0.895	0.993

### 3.3 NIH 3T3 fibroblast cell viability

The viability of NIH 3T3 fibroblast cells in the proximity of the core-shell nanofibers w and w/o drugs is demonstrated in Figure 4C. Both classes of the core-shell nanofibers show a comparable cell viability to the control group, i.e., TCPS, after 24, 48, and 72 h. There is no meaningful impact of the incorporated drugs on cell viability, despite the initial burst release of some part of both LEV and QS in 2 h. The comparable cell viability of the core-shell nanofibers with the control implies the supportive role of the nanofibers towards cell proliferation. Apart from the biomimetic nanofibrous structure of the mats that could encourage the cells for adhesion and proliferation, PLGA could partially degrade within the culture medium, thereby promoting the cell activities. Through PLGA degradation, lactate is released that can potentially provoke the proliferation of endothelial and fibroblast cells (Chereddy et al., 2013; Chereddy et al., 2015). The lactate induced collagen deposition by cultured fibroblasts is an established fact (Green and Goldberg, 1964) and the increased amount of lactate in healing wounds drives collagen synthesis and wound repair (Hunt et al., 1978). Lactate activates collagen prolylhydroxylase, which is an enzyme that governs procollagen hydroxylation and collagen maturation in fibroblasts, thereby enhancing collagen synthesis (Porporato et al., 2012). In contrast to PLGA, PEO with the high average M_v_ of 900,000 and CS as bonded with LEV and PEO (similar to cross-linked CS) cannot biodegrade (Hong et al., 2007; Lei et al., 2022) within 3 days of cell culture and contribute to cell viability. Therefore, the main material of the core-shell nanofibers that plays a determining role in fibroblast cell viability is PLGA. In general, the cell viability data suggest that the core-shell nanofibers w and w/o LEV and QS are of high potential for wound healing, particularly given their bacteriostatic and antioxidant activity.

### 3.4 *In vivo* wound healing efficiency

**FIGURE 5:**
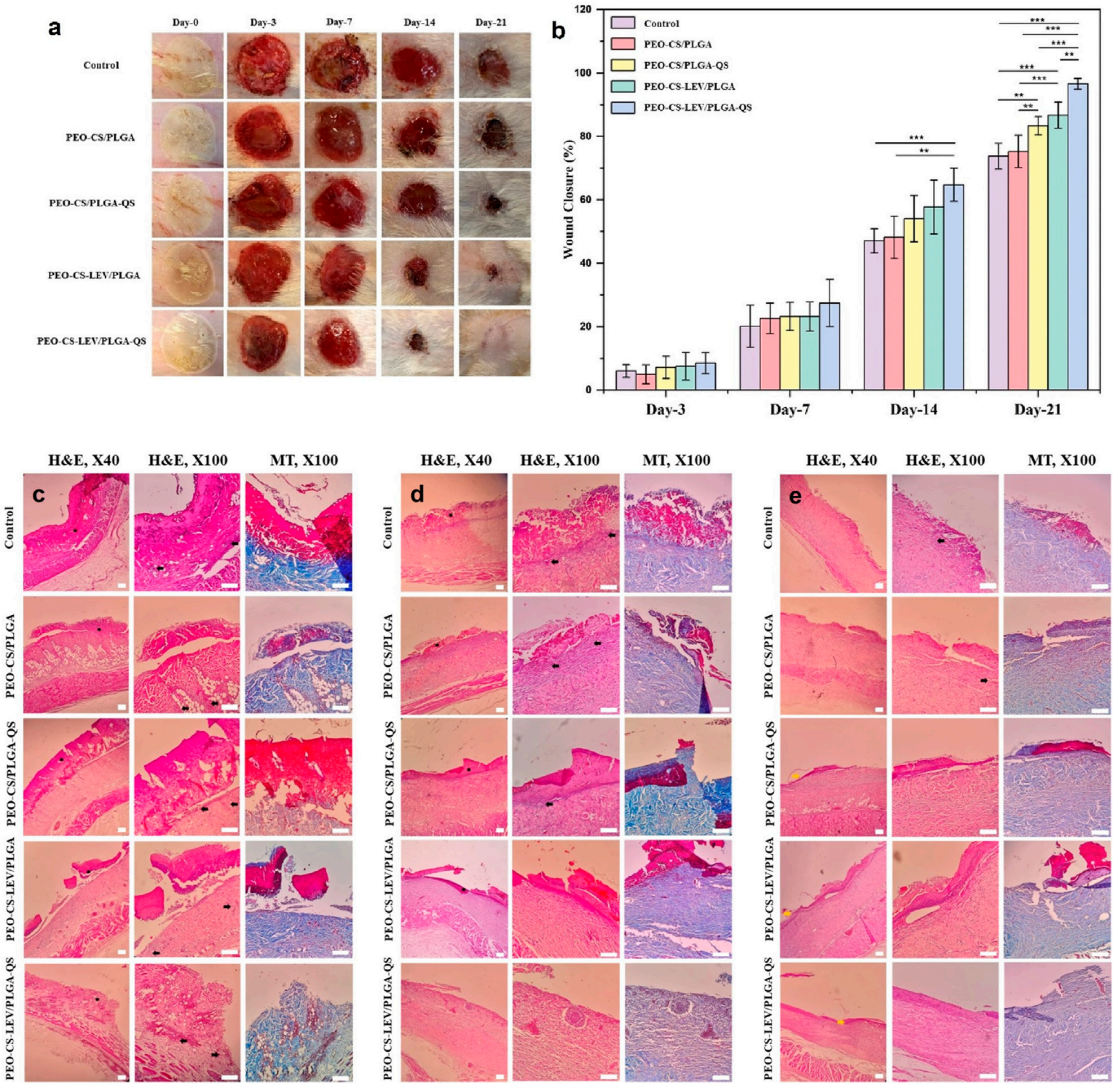
**(A)** *In vivo* wound healing efficiency and **(B)** wound closure percentage of the PEO-CS-LEV/PLGA-QS core-shell nanofibrous dressing compared to semi-drug (either LEV or QS) loaded and unloaded core-shell nanofibrous dressings over a 3-week treatment period. Evidently, thanks to co-delivery of QS and LEV, the burn wound treated with the drug loaded core-shell nanofibrous dressing shows a larger wound healing efficiency reflected in complete wound closure after 21 days. The wound closure percentage induced by the drug loaded core-shell nanofibrous dressing prevails over that made by the unloaded core-shell nanofibrous dressing and control on the 14th and 21st days of treatment (**: *p* < 0.01, ***: *p* < 0.001). Haematoxylin-eosin (H&E) and Masson’s trichrome (MT) stained histopathological images of the burn wound tissues after treatment with the PEO-CS-LEV/PLGA-QS core-shell nanofibers for 7 **(C)**, 14 **(D)**, and 21 **(E)** days (dark star, dark arrow, and yellow arrow mark crusty scab, inflammation, and re-epithelialization, respectively) (scale bars represent 200 µm).

The core-shell nanofibrous dressings w and w/o LEV and QS were tested *in vivo* to verify that the sustained release of antibacterial and antioxidant drugs could prevent infection of burn wounds while promoting wound healing. Figure 5A illustrates the images of the burn wounds treated with the core-shell nanofibrous dressings on third, seventh, 14th, and 21st days post-treatment. While in the control (gauze treated) group and the drug free core-shell nanofiber group, wound healing delayed, the burn wound treated with the PEO-CS-LEV/PLGA-QS nanofibrous dressing healed with the fastest rate. Thanks to the co-delivery of QS and LEV, the largest wound closure rate was achieved until day 21. QS is a well-known flavonoid compound with anti-inflammatory and antioxidant properties. QS can provoke wound healing via mediating inflammation, increasing the proliferation rate of fibroblast cells, and lowering the immune cells infiltration (Chittasupho et al., 2021). Additionally, LEV is a third-generation fluoroquinolone antibiotic that can inhibit gram-negative, gram-positive, and anaerobic bacteria. The prolonged local delivery of antimicrobials, e.g., LEV can prevent wound infection while improving wound healing (Hassani et al., 2022). According to Vipin et al. (Vipin et al., 2020), in combination with antibiotics, QS can synergistically offer enhanced therapeutic effects and significantly inhibit biofilm formation, compared to monotherapy. Figure 5B shows the wound closure percentage of the burn wounds treated with different classes of the core-shell nanofibrous dressings. Other than the third and seventh days, there is a significant difference in wound closure percentage of the wounds treated with the drug loaded core-shell nanofibers with those treated with the drug free core-shell nanofibers (*p* < 0.01 on day 14 and *p* < 0.001 on day 21) and control (gauze treated wound) (*p* < 0.001 on day 14 and 21).

Similarly, Ajmal et al. (Ajmal et al., 2019) have reported that Ciprofloxacin hydrochloride and QS loaded polycaprolactone electrospun nanofibers can stimulate the wound healing process, thanks to the antimicrobial and antioxidant properties of the incorporated drugs. The inhibition of microbial infection and oxidative damage to fibroblasts by excess reactive oxygen species (ROS) lead to improved wound healing conditions.

### 3.5 Histopathology of the burn wounds treated with the core-shell nanofibrous dressing

The histopathological images obtained through H&E and Masson’s trichrome staining further confirm the wound healing efficiency of the drug loaded core-shell nanofibrous dressings after 7, 14, and 21 days (Figures 5C–E, respectively). The H&E images (Figure 5E) show that the tissues treated with the semi or full drug-loaded core-shell nanofibers possess an integrated epidermis compared to the other treatment groups. Masson’s trichrome staining was used to assess the deposition and reconstruction of collagen fibers in the regenerated skin. Collagen deposition at the dermis layer of the tissues treated with PEO-CS-LEV/PLGA-QS core-shell nanofibrous dressing revealed densely distributed collagen and a complete healing process compared to the other groups, as shown in Figure 5E. On the other hand, the nanofibrous dressing w/o drug treated group and control group displayed few areas in the dermis layer where collagen was not completely reconstructed.

The wound healing process unites several overlapping phases of homeostasis, inflammation, proliferation/granulation, and remodeling/maturation (Negut et al., 2020). Excessive inflammation and diminished angiogenesis pose significant challenges to the process of wound healing and skin regeneration. Consequently, addressing inflammation emerges as a pivotal factor that must be carefully considered. Our results show that while on day 7, the signs of inflammation and formation of crusty scab were clearly observed in all the wound tissues treated with the different nanofibrous dressings, these signs almost vanished on day 14 in the tissues treated with the semi or full drug-loaded core-shell nanofibers. Comparatively, the control group was still inflamed and largely covered by crusty scab on day 14. Thanks to the anti-inflammatory effect of QS and antibacterial activity of LEV, the wounds treated with PEO-CS-LEV/PLGA-QS nanofibrous dressings exhibited no signs of inflammation on day 14. Lu et al. (Lu et al., 2022) have reported that bacterial colonization and endotoxin production in a wound site can result in prolonged inflammatory phase and thus delay wound healing. In this regard and aligned with our findings, Suhaeri et al. (Suhaeri et al., 2018) have indicated that antimicrobial wound dressings can lower bacterial toxin-induced inflammation and consequently facilitate wound healing. On day 21, re-epithelialization in the wounds treated with the semi and full drug loaded core-shell nanofibrous dressings was evident. Most notably, the PEO-CS-LEV/PLGA-QS nanofibrous dressing could significantly promote re-epithelialization in the wound tissue as reflected in the formation of discernible epidermis layers.

## 4 Conclusion

Chronic wounds are a significant burden on patients and healthcare systems worldwide. These challenging medical crises are multifaceted and require treatments that address several therapeutic needs simultaneously. For instance, chronic wounds are highly inflamed and susceptible to infection. In the present study, we developed novel PEO-CS-LEV/PLGA-QS core-shell nanofibers using co-axial electrospinning technique. These core-shell nanofibers could effectively co-deliver LEV and QS, i.e., two therapeutic compounds with antibacterial and antioxidation activities, to burn wounds. As validated by *in vitro* and *in vivo* studies, the drug loaded core-shell nanofibrous dressings could promote wound healing rate, that might be a consequence of lowered bacterial load and oxidative stress within the treated wounds. It is crucial to emphasize that on day 21, there were no indications of blood inflammatory cells—such as neutrophils, lymphocytes, or macrophages—nor were there any signs of abscess or exudates like pus in the tissues treated with either semi or fully drug-loaded core-shell nanofibers. Conclusively, our study could develop PEO-CS/PLGA core-shell nanofibers loaded with QS and LEV as a promising wound healing material and validate its therapeutic potentials *in vivo*.

## Data Availability

The original contributions presented in the study are included in the article/Supplementary Material, further inquiries can be directed to the corresponding authors.
