# Supplementary material for: Levofloxacin loaded poly (ethylene oxide)-chitosan/quercetin loaded poly (D,L-lactide-co-glycolide) core-shell electrospun nanofibers for burn wound healing
Source: Front Bioeng Biotechnol. 2024 Mar 28;12:1352717. doi: 10.3389/fbioe.2024.1352717 (PMC11007221; doi:10.3389/fbioe.2024.1352717)
Supplement: Supplementary file 1 [file DataSheet1.docx]

**Supplementary information**

**Levofloxacin loaded poly (ethylene oxide)-chitosan/quercetin loaded poly (D,L-lactide-co-glycolide) core-shell electrospun nanofibers for burn wound healing**

Mahshid Monavari^1^, Razieh Sohrabi^1^, Hamidreza Motasadizadeh^2^, Mehran Monavari^3^, Yousef Fatahi^4,5^, Negin Mousavi Ejarestaghi^6^, Miguel Fuentes-Chandia^7^, Aldo Leal-Egaña^8^, Mohammad Akrami^1,9,*^, Shahin Homaeigohar^10, *^

^1^ Department of Pharmaceutical Biomaterials and Medical Biomaterials Research Center, Faculty of Pharmacy, Tehran University of Medical Sciences, Tehran, Iran.

([Mahshid.monavari@gmail.com](mailto:Mahshid.monavari@gmail.com) & [Sohrabie.nas85@gmail.com](mailto:Sohrabie.nas85@gmail.com)).

^2^ Dental Research Center, Dentistry Research Institute, Tehran University of Medical Sciences, Tehran, Iran ([h.motasadi@gmail.com](mailto:h.motasadi@gmail.com)).

^3^ Section eScience (S.3), Federal Institute for Materials Research and Testing, Berlin, Germany.

(mehran.monavari@bam.de)

^4^ Nanotechnology Research Centre, Faculty of Pharmacy, Tehran University of Medical Sciences, Tehran, Iran ([youseffatahi@gmail.com](mailto:youseffatahi@gmail.com)).

^5^ Department of Pharmaceutical Nanotechnology, Faculty of Pharmacy, Tehran University of Medical Sciences, Tehran, Iran.

^6^ Department of Pharmaceutics, Faculty of Pharmacy, Tehran University of Medical Sciences, Tehran, Iran ([negin.mou.e@gmail.com](mailto:negin.mou.e@gmail.com)).

^7^ Department of Biology, Skeletal Research Center, Case Western Reserve University, Cleveland, OH, United States ([miguel.angel.fuentes@hotmail.com](mailto:miguel.angel.fuentes@hotmail.com)).

^8^ Institute for Molecular Systems Engineering and Advanced Materials, Heidelberg University, Germany ([aldo.leal@uni-heidelberg.de](mailto:aldo.leal@uni-heidelberg.de)).

^9^ Institute of Biomaterials, University of Tehran & Tehran University of Medical Sciences (IBUTUMS) Tehran, Iran (m-akrami@sina.tums.ac.ir).

^10^ School of Science and Engineering, University of Dundee, Dundee DD1 4HN, UK (Shomaeigohar001@dundee.ac.uk).

***** Correspondence: m-akrami@sina.tums.ac.ir (M.A.) and [Shomaeigohar001@dundee.ac.uk](mailto:Shomaeigohar001@dundee.ac.uk) (S.H.)


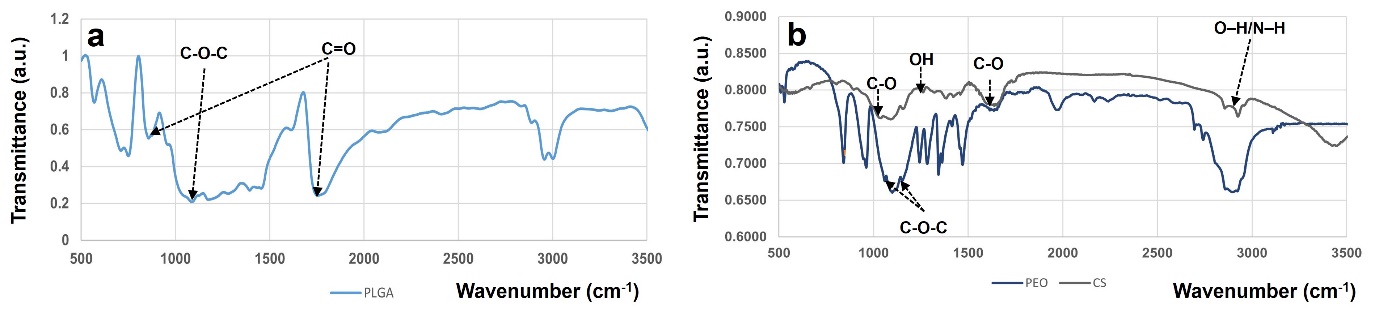


**Figure S1.** FTIR spectra for: a) PLGA nanofibers, and b) PEO and CS nanofibers. The characteristic functional groups of each polymer have been marked at their respective band location.


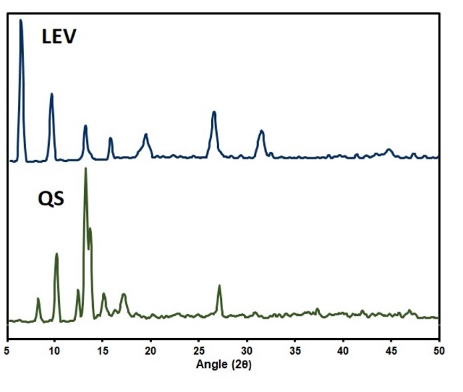


**Figure S2.** XRD spectra of LEV and QS, implying their highly crystalline nature.


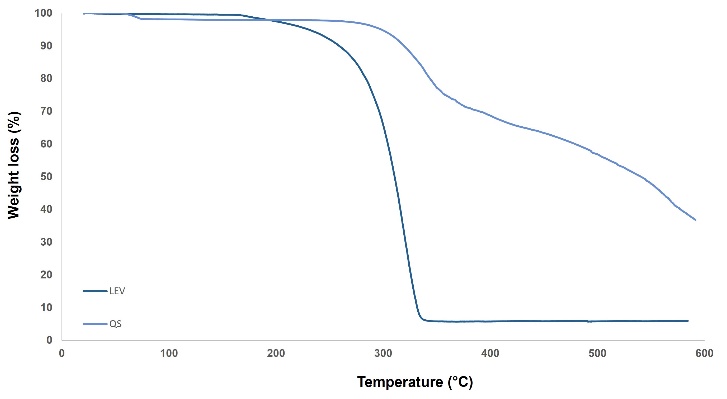


**Figure S3.** TGA profile of LEV and QS. LEV undergoes a major weight loss at 192 – 361 °C because of thermal degradation and decomposition [1]. QS undergoes two major weight losses in the temperature ranges of 65–103 °C and 285 – 410 °C due to evaporation of the adsorbed water and thermal degradation, respectively [2].

**Table S1.** Mechanical properties of different classes of the core-shell nanofibers w and w/o LEV and QS.

|  | Tensile strength (MPa) | Elongation at break (%) | Young's Modulus (MPa) |
| --- | --- | --- | --- |
| PEO-CS/PLGA | 3.05 | 28.6 | 0.25 |
| PEO-CS/PLGA-QS | 3.14 | 37.9 | 0.26 |
| PEO-CS-LEV/PLGA | 3.32 | 26.3 | 0.37 |
| PEO-CS-LEV/PLGA-QS | 3.37 | 30.4 | 0.33 |

[1] J. Jalvandi, M. White, Y. Gao, Y.B. Truong, R. Padhye, I.L. Kyratzis, Polyvinyl alcohol composite nanofibres containing conjugated levofloxacin-chitosan for controlled drug release, Materials Science and Engineering: C 73 (2017) 440-446.

[2] L. Zhang, X. Yang, S. Li, W. Gao, Preparation, physicochemical characterization and in vitro digestibility on solid complex of maize starches with quercetin, LWT-Food Science and Technology 44(3) (2011) 787-792.
